# Book Notices

**Published:** 1869-11

**Authors:** 


### Book Notices.

The Physical Life of Woman: Advice to Maiden, Wife, and Mother. By Geo. H. Napheys, A.M., M.D.; Author of Compendium of Modern Therapeutics, etc. Philadelphia: Geo. McLean, 719 Sansom Street. 1869.

This is a neatly bound volume of 250 pages, written in a plain and pleasing style, well calculated both to please and instruct. There is nothing of the *sensational* or imaginative character in it. On the contrary, its teachings are in strict accordance with scientific facts and good sense. Though designed specially for females, yet a careful perusal would be productive of much benefit to both sexes. Price $1.50.

---

A Course of Practical Chemistry, Arranged for the Use of Medical Students. By WILLIAM ODLING, M.B., F.R.S.; Fellow of Royal College of Physicians; Lecturer on Chemistry at St. Bartholomew's Hospital. With Illustrations. From the Fourth and Revised London Edition. Philadelphia: HENRY C. LEA. 1869. Pp 261. Price $2.

This is an excellent manual of practical chemistry, and is particularly well adapted to the wants of the medical student, who should regard a good knowledge of practical or analytical chemistry as a necessary part of his professional education. For sale by S. C. Griggs & Co., Chicago.

---

Reports on the Diseases of Cattle in the United States, made to the Commissioner of Agriculture, with Accompanying Documents. Washington, 1869.

We have received this volume direct from the Department of Agriculture, at Washington. It is an octavo volume of 190 pages, well bound; and contains a full and interesting report on the lung plague, or Texas cattle disease, giving its history, mode of spread, nature, and management, by Professor John Gamgee, M.D. The same author has two other reports; one on the "Ill Effects of Smutty Corn on Cattle," and the other "On the Splenic or Periodic Fever of Cattle." These papers occupy nearly 160 pages of the volume. The remainder is occupied by "General Remarks on the Cattle Diseases Reported on;" "Remarks on the Exodes Bovis," by C. N. Riley, St. Louis, Mo.; on "The Fungi of Texas," by H. W. Ravenal, of South Carolina; and a "Report of the Results of Examinations of the Fluids of Diseased Cattle, with Reference to the Presence of Cryptogamic Growths," by Drs. Billings and

Curtis, of the United States Army. All these are topics of very great importance, and are treated by men in the highest degree qualified for the work.

It is greatly to be regretted that the Commissioner of Agriculture was obliged to omit numerous and highly important illustrations for want of funds. We trust that among the first acts of the coming Congress will be an appropriation sufficient to meet the expenses of a new edition, with all the illustrations which the importance of the subjects treated require.

---

The Mechanism of Dislocation and Fracture of the Hip. With the Reduction of the Dislocations by the Flexion Method. By HENRY J. BIGELOW, M.D., Prof. Surgery and of Clinical Surgery in the Medical School of Harvard University; Surgeon of the Massachusetts General Hospital, etc., etc. With illustrations. Philadelphia: HENRY C. LEA. 1869. Pp 150. For sale by W. B. KEEN & COOKE, Chicago.

This is an elegantly published volume, containing a complete presentation of the present state of surgical knowledge, in regard to dislocations and fractures of the hip, with accurate illustrations. It is a valuable addition to the surgical literature of our profession.

---

Physicians' Visiting List for 1870—Nineteenth year of its Publication. Philadelphia: LINDSAY & BLAKISTON. Sold by all Booksellers and Druggists.

This pioneer still holds its reputation as one of the most convenient pocket visiting lists and account books that the physician can use. The copies for 1870 are issued, and in the hands of booksellers in good time.

---

Transactions of the Illinois State Medical Society; Nineteenth Anniversary Meeting, held in Chicago, May 18th, 19th, 20th, 1869.

This is a well printed volume, in paper cover, 160 pages. Besides the record of proceedings, it contains ten reports and papers of more or less interest and value, and a list of names of members of the Society. Secretary, T. D. Fitch, M.D., Chicago.

---

Minutes of the First Annual Meeting of the Nebraska State Medical Society, held at Nebraska City, June 1st, 2d, 1869.

This is a very neatly printed pamphlet of 24 pages, containing the record of proceedings, and brief reports from the Society, the Publication Committee, the section on surgery, the section on obstetrics, together with a copy of the constitution and by-laws of the Society. It indicates a good spirit in the profession of this new State. Secretary, S. D. Mercer, M.D., of Omaha.

